# Molecular Docking and Molecular Dynamics Studies of Antidiabetic Phenolic Compound Isolated from Leaf Extract of *Englerophytum magalismontanum* (Sond.) T.D.Penn.

**DOI:** 10.3390/molecules27103175

**Published:** 2022-05-16

**Authors:** Oyinlola Oluwunmi Olaokun, Sizakele Annousca Manonga, Muhammad Sulaiman Zubair, Saipul Maulana, Nqobile Monate Mkolo

**Affiliations:** 1 Department of Biology, School of Science and Technology, Sefako Makgatho Health Science University, Molotlegi Street, Ga-Rankuwa, Pretoria 0204, South Africa; sizakele.manonga@gmail.com (S.A.M.); nqobile.mkolo@smu.ac.za (N.M.M.); 2 Natural Product Research Group, Department of Pharmacy, Faculty of Science, Tadulako University, Palu-Central Sulawesi 94118, Indonesia; sulaimanzubair@untad.ac.id (M.S.Z.); saifulmaulana011@gmail.com (S.M.)

**Keywords:** *Englerophytum magalismontanum* leaf, naringenin, α-amylase, α-glucosidase, molecular docking, molecular dynamics

## Abstract

*Englerophytum magalismontanum*, a medicinal plant with ethnopharmacology use, has a dearth of information regarding its antidiabetic properties. This study evaluated the crude methanol leaf extract of *E. magalismontanum* and its fractions for total phenolic content, antioxidant activity, and digestive enzymes (α-amylase and α-glucosidase) inhibitory activity using standard methods. The total phenolic content (56.53 ± 1.94 mg GAE/g dry extract) and DPPH Trolox antioxidant equivalent (TAE) (1.51 ± 0.66 µg/mL) of the methanol fraction were the highest among the fractions. The IC_50_ values of the methanol fraction against α-amylase (10.76 ± 1.33 µg/mL) and α-glucosidase (12.25 ± 1.05 µg/mL) activities were also high. Being the most active, the methanol fraction was subjected to bio-assay guided column chromatography-based enzyme inhibition to obtain a pure compound. The phenolic compound isolated and identified as naringenin inhibited α-amylase and α-glucosidase with IC_50_ of 5.81 ± 2.14 µg/mL and 4.77 ± 2.99 µg/mL, respectively. This is the first study to isolate naringenin from *E. magalismontanum* extract. The molecular docking and molecular dynamics studies demonstrated naringenin as a promising lead compound in comparison to acarbose for the treatment of diabetes through the inhibition of α-glucosidase activity.

## 1. Introduction

Diabetes mellitus is one of the major chronic diseases present in civilization, affecting millions of people worldwide [1]. Over the last 10 years, it has been increasing rapidly in both middle- and low-income countries [2,3]. Worldwide, the prevalence of diabetes among adults over 18 years of age has risen from 4.7% in 1980 to 8.8% in 2014 [2,3]. In 2017, an estimated 4.0 million deaths were related to diabetes and its complications [4]. The estimated cost worldwide due to this disease is about 673 billion dollars, with 80% of this attributed to rich economies and only 20% to poor economies [2]. The control measures and problems related to diabetes mellitus are well documented by many researchers [3,5,6,7].

Although several therapeutic options are available for the management of diabetes mellitus, treatment is associated with adverse side effects, complications, and the inability to prevent the deterioration of pancreatic β-cells [6,8,9]. As a result, there is a need for effective treatments, and the use of plant-based products is proposed as an alternative treatment method [10]. Plants rich in phytochemicals such as flavonoids, polyphenols, and organic acids have exhibited antidiabetic potential in vitro by reducing intestinal glucose absorption, increasing insulin secretion of pancreatic cells, and glucose uptake of muscle cells [11]. These phytochemicals, in addition, possess antioxidant potentials which ameliorate the oxidative stress induced by prolonged hyperglycaemia and inflammation [11]. Obesity is an inflammatory condition that secretes cytokines that confer insulin resistance in the liver, skeletal muscle, and endothelial tissues leading to the clinical expression of type II diabetes [12,13]. Hence, for this study, *Englerophytum magalismontanum* was investigated as a potential candidate for antidiabetic compounds.

*Englerophytum magalismontanum* (Sond.) T.D.Penn (Sapotaceae) in traditional medicine is used to treat ailments such as infertility, coughs, colds, bronchial problems, fever, asthma, abdominal pains, chronic coughs, pleurisy, and skin disorders [14,15,16,17]. It is one of the South African plant species with ethnobotanical uses for inflammatory conditions [18]. *E. magalismontanum* is a small to medium-sized evergreen tree that varies in height from 3 to 10 m depending on the habitat. It is found in the riverine forest fringes in Gauteng, Northwest, Mpumalanga, Limpopo, and the northern parts of KwaZulu-Natal, South Africa. The leaves are often crowded near the end of the branchlets; young leaves tightly fold upward along the midrib with russet hairs, oblanceolate to obovate-elliptic, usually, 7–14 × 2–5 m, is a glossy dark green [15].

Currently, there is a dearth of information on aspects of glucose metabolism in this plant and the characterization of the phytochemicals of the extract responsible for this health benefit and its relationship, if any, with antioxidant activity. The first objective of this study was to investigate the crude methanol extract of *E. magalismontanum* and the solvent-solvent fractionated or partitioned fractions of the crude methanol extract for total polyphenol content, antioxidant activity, and α-amylase and α-glucosidase inhibitory activities. The second objective was to isolate and identify the active phenolic compound obtained through bioassay-guided column chromatography fractionation and to investigate its binding interactions with α-glucosidase by molecular docking and molecular dynamics studies.

## 2. Results and Discussion

### 2.1. Plant Extraction and Biological Activities of Crude Methanol Extract and Its Fractions

#### 2.1.1. Plant Extraction and Solvent-Solvent Fractionation of Crude Extract

The dried methanol crude extract of *E. magalismontanum* leaves was subjected to solvent-solvent fractionation with solvents of different polarities. The methanol fraction extracted the highest amount (89.0 g) of extractable material representing a yield of 38.7%, while the n-hexane extracted the lowest mass (11.7 g) of extractable component representing a yield of 5.1%. The percentage yield of the extractable material was calculated from the formula: the weight of dry extract divided by the weight of the dry crude extract multiplied by one hundred. Solvent–solvent fractionation is one of the first techniques often utilized in the process of obtaining pure natural products from complex extracts [19,20]. With this method, various polarities of solvents are applied in the liquid technique to extract the phytochemical groups of plants [21]. The efficiency of this fractionation technique is dependent on many conditions, including fluctuations in solvent, temperatures, and time [21].

#### 2.1.2. Antioxidant Activity and TPC of Crude Extract and Its Fractions

The antioxidant activity was determined by the DPPH free radical scavenging activity of *E. magalismontanum* crude methanol extract, and the fractions were expressed as the Trolox antioxidant equivalent (TAE) (Table 1). The TAE of the DPPH radical scavenging activity of the methanol fraction was the strongest (1.51 ± 0.66 µg/mL) though not significantly different from that of the crude methanol extract (1.66 ± 0.63 µg/mL). For the antioxidant activity evaluation of plants, several methods have been proposed, including the total antioxidant activity, metal chelation, radical scavenging (DPPH) effects, and the reducing power, as well as activities that are destructive to active oxygen species, such as the superoxide anion radical, hydroxyl radical, and hydrogen peroxide [22,23]. In this study, the DPPH was adopted to determine the antioxidant capacity of the crude extract and its fractions using the DPPH assay. This is due to its measurement simplicity, short experimental time, and the use of an inexpensive spectrophotometer [24].

The total phenolic content (TPC) calculated from the regression equation of the gallic acid calibration curve for the methanol fraction (56.53 ± 1.94 mg GAE/g dry extract) was significantly higher (*p* < 0.05) than for the crude methanol extract (49.78 ± 0.40 mg GAE/g dry extract) (Table 1). The results of the antioxidant activity and TPC indicated that the phenolic compounds of the crude methanol extract and its fractions are likely to be responsible for the DPPH free radical scavenging activity. The unique structure and high tendency of phenolic compounds for metal chelation and their redox properties allow them to act as reducing agents, hydrogen donators, and singlet oxygen quenchers which could make them exhibit antioxidant and antidiabetic potentials [25,26,27].

#### 2.1.3. α-Amylase and α-Glucosidase Inhibitory Activity of Crude Extract and Its Fraction

The α-amylase and α-glucosidase inhibitory activities of the *E. magalismontanum* crude methanol extract and fractions expressed as IC_50_ are presented in Table 2. The crude methanol extract and its fractions inhibited the activities of α-amylase and α-glucosidase to various extents. The methanol fraction (MF) was the most active among the fractions, with IC_50_ of 10.76 ± 1.33 µg/mL and 12.27 ± 1.55 µg/mL against the activities of α-amylase and α-glucosidase, respectively, but not significantly different from the crude extract. The inhibition of the key enzymes involved in the hydrolysis of dietary starch, α-amylase for carbohydrate breakdown and α-glucosidase for intestinal absorption, may be an important strategy in the management of postprandial hyperglycaemia [28,29]. Plant extracts rich in phenolic compounds are reported to possess the ability to inhibit the activity of enzymes, including those of α-amylase and α-glucosidase [29,30]. However, the results of the TPC and enzyme inhibition by *E. magalismontanum* crude methanol extract fractions and sub-fractions indicated that TPC alone may not be involved in the enzyme inhibition and that other factors may be involved [31].

### 2.2. Isolation and Identification of the Structure of Active Compound

**Figure 1:**
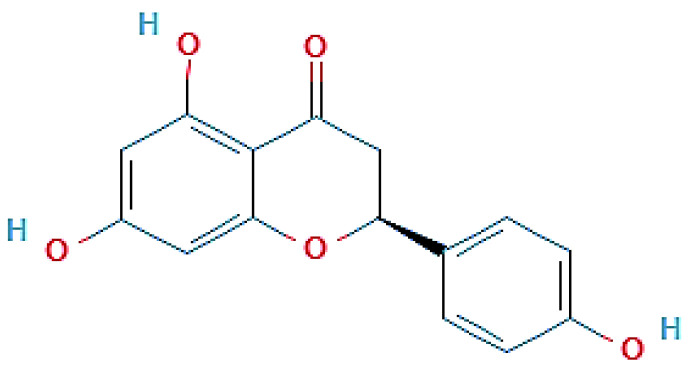
Chemical structure of naringenin (Álvarez-Álvarez et al. [34]).

The column chromatography fractionation of the most active fraction (MF) yielded four sub-fractions (MF1-4) when pooled together, and these were subjected to enzyme assays. The result of the α-amylase and α-glucosidase inhibitory activity of the sub-fractions of MF and the isolated compound are presented in Table 2. The inhibition of sub-fraction MF3 against the activities of α-amylase (12.49 ± 0.96 µg/mL) and α-glucosidase (10.19 ± 1.04 µg/mL), though high, was not significantly different from those of the other sub-fractions. Furthermore, the column chromatographic fractionation of MF3 produced two sub-fractions (MF3-1 and MF3-2) when pooled together. While the IC_50_ of sub-fraction MF3-1 was (8.79 ± 2.23 µg/mL) and (6.88 ± 0.79 µg/mL) against α-amylase and α-glucosidase, respectively, that of sub-fraction MF3-2 was (8.71 ± 1.42 µg/mL) and (7.84 ± 2.61 µg/mL) against α-amylase and α-glucosidase, respectively. The compound obtained after subjecting sub-fractions MF3-1 and MF3-2 to preparative TLC fractionation inhibited the activities of α-amylase and α-glucosidase with IC_50_ of 5.81 ± 2.14 µg/mL and 4.77 ± 2.99 µg/mL, respectively. The identity of the isolated compound was elucidated by NMR spectroscopy. The analysis of the NMR data (Table 3) together with Figures S1–S4 in the Supplementary Material suggested that the isolated compound was a flavanone and had the structure of naringenin (Figure 1) [32] by comparison with previously reported data [33,34,35,36]. To the best of our knowledge, this is the first study to isolate naringenin from leaf extracts of *E. magalismontanum*. Naringenin is reported to exhibit numerous biological activities and potential health benefits [37,38]. It possesses antioxidant, antiproliferative, antitussive, and hepatoprotective properties. Several investigations suggest that naringenin supplementation is beneficial for the treatment of obesity, diabetes, hypertension, and metabolic syndrome [39,40]. However, this effect on obesity, diabetes, hypertension, and metabolic disorder remains to be fully established. It is known that the inhibition of α-glucosidase and α-amylase decreases postprandial blood glucose levels and delays glucose absorption, making it a suitable treatment strategy for type II diabetes.

### 2.3. In Silico Activity of Acarbose and Naringenin against α-Glucosidase Receptor

#### 2.3.1. Molecular Docking of Acarbose and Naringenin

The molecular docking study was meant to analyze the binding mode of acarbose and naringenin in the active site of the α-glucosidase receptor based on binding energy and molecular interactions. The results showed that acarbose had a higher binding affinity to bind on the α-glucosidase receptor with lower binding energy (−7.9 kcal/mol) than naringenin (−7.0 kcal/mol) (Table 4). Furthermore, the binding mode of each compound was further analyzed for molecular interactions. Human α-glucosidase is known to catalyze the cleavage of short α-(1→4) oligosaccharide units [41]. This process is facilitated by three amino acid residues (ASP203, ASP327, and ASP542) as the catalytic site of the receptor.

**Figure 2:**
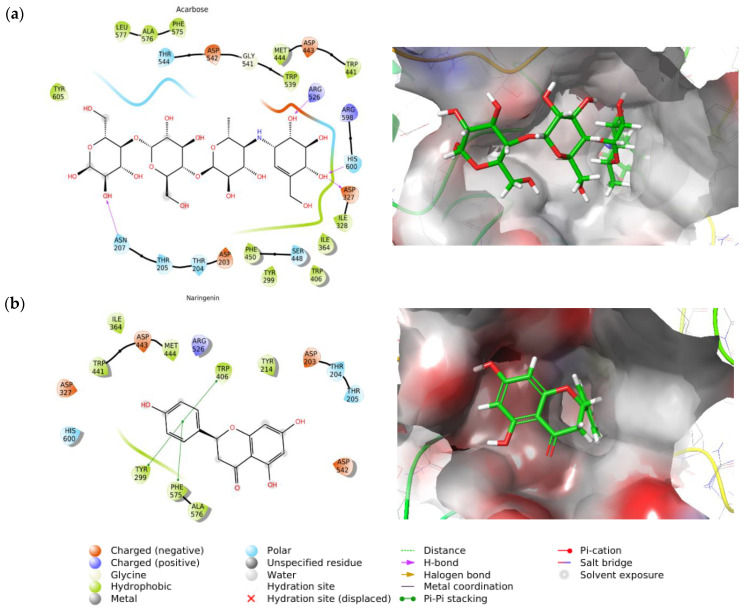
Molecular interactions of: (**a**) acarbose and (**b**) naringenin, with α-glucosidase receptor.

The result of the molecular interactions (Figure 2) showed that acarbose formed hydrogen bonds with ASP327, ARG526, and HIS600 through hydroxyl groups. Meanwhile, hydrophobic interactions occurred with TYR299, ILE328, ILE364, TRP406, TRP441, MET444, PHE450, TRP539, PHE575, ALA576, LEU577, and TYR605. These interactions possessed an increasing electronic affinity that implicates acarbose as having lower binding energy than naringenin. Although naringenin possessed high binding energy, this might have been an interaction with the catalytic sites (ASP203, ASP327, and ASP542) that inhibited the receptor’s (α-glucosidase) activity even without forming a hydrogen bond. This interaction indicated that naringenin might inhibit α-glucosidase activity as well. Similar to acarbose, naringenin generated hydrophobic interactions with numerous amino acids, i.e., ILE364, TRP441, MET444, TYR214, ALA576, as well as π-π interactions with TYR299, TRP406, and PHE575. These results suggest naringenin as a promising lead compound for the treatment of diabetes mellitus through the inhibition of α-glucosidase.

#### 2.3.2. Molecular Dynamics of Acarbose and Naringenin

**Figure 3:**
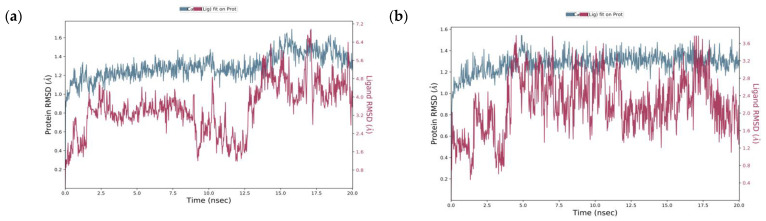
Root mean square deviation (RMSD) of alpha carbon (Cα) of the receptor (blue) and ligand (red) of (**a**) acarbose and (**b**) naringenin, on the α-glucosidase binding site during the 20 ns simulation.

Molecular dynamics (MD) was performed to validate the accuracy of the docking results and explore the detailed molecular interactions. According to the results regarding the structure of molecular docking, acarbose and naringenin were buried in the α-glucosidase binding site. In the MD studies, root mean square deviation (RMSD) was used as an indicator that evaluates the average conformational change of an atom concerning reference conformations [42]. The RMSD of acarbose was initially at 0.9 Å, which then fluctuated up to 4 Å in 2.5 ns (Figure 3). Thereafter, the system was eventually stabilized until 12.5 ns, which was indicated by the reduction in the RMSD value to 1.6 Å. Subsequently, RMSD fluctuated up again over 4 Å and remained consistent until the simulation was over. Although the RMSD of acarbose fluctuated during the simulations, the divergence with the protein RMSD was still under the acceptable range of 1–3 Å (2.21 Å), thus reflecting a non-significant difference. Unlike acarbose, the RMSD of naringenin commenced at 1.2 Å, which fluctuated until 3 ns. After 5 ns of simulation time, the naringenin was stabilized over the final simulations along 20 ns trajectories with the RMSD plot of the naringenin—α-glucosidase complex at 2.20 Å, suggesting that naringenin is stably bound and not diffused away from the α-glucosidase active site.

**Figure 4:**
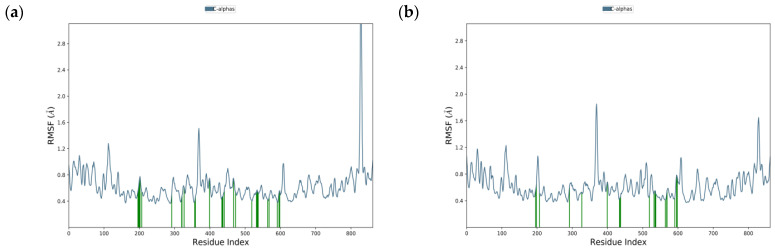
Root mean square fluctuation (RMSF) plotting (blue line) of: (**a**) acarbose and (**b**) naringenin, with specific residues of α-glucosidase (green line) during 20 ns simulation.

The MD studies could be helpful in assessing the stability of the interaction between the ligand and the specific amino acids on the receptor’s binding site by computing the root mean square fluctuation (RMSF). RMSF may also evaluate the protein’s flexibility region by describing the conformational changes in the protein chain along simulation times. For the root mean square fluctuation (RMSF) plot (Figure 4), both compounds had RMSF valued below 2 Å except for the interaction between acarbose and amino acid SER836, which showed a high RMSF value of 6.091 Å.

To analyze the interaction of the ligand-receptor on the catalytic sites, the green line on the RMSF plots was used to represent the specific amino acids that interact with the compounds. Acarbose has 32, while naringenin has only 21 interactions with numerous specific amino acids in the α-glucosidase active site. This result showed that acarbose interacts with more amino acids than naringenin, which implies an improvement in the stability of the acarbose–glucosidase complex. Acarbose was found to interact with the catalytic sites ASP203, ASP327, and ASP542 with RMSF values of 0.531 Å, 0.424 Å, and 0.591 Å, respectively. Meanwhile, naringenin barely interacted with ASP 327 and merely with ASP203 (0.598 Å) and ASP542 (0.528 Å) along 20 ns of simulation time. This result suggests that naringenin is also stable when interacting with catalytic sites, as indicated by the lower RMSF value on the amino acid of ASP203 and ASP542, which represented minor conformational change.

**Figure 5:**
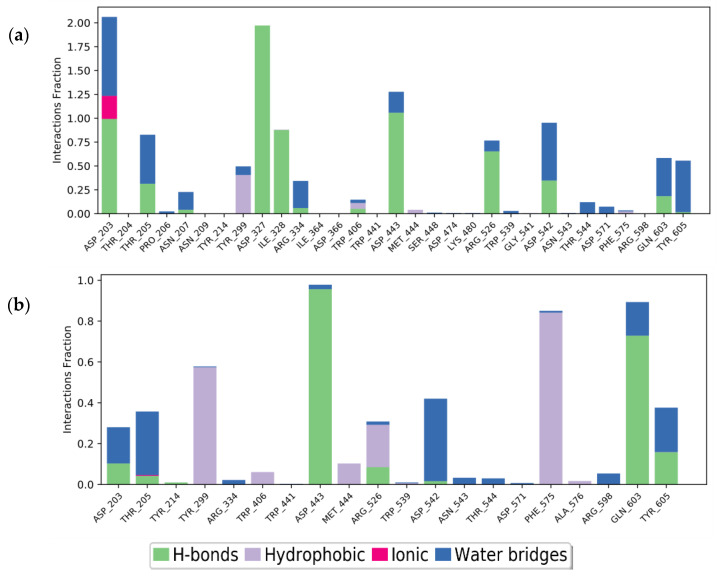
Protein-ligand interaction of: (**a**) acarbose and (**b**) naringenin, after 20 ns MD simulation time.

**Figure 6:**
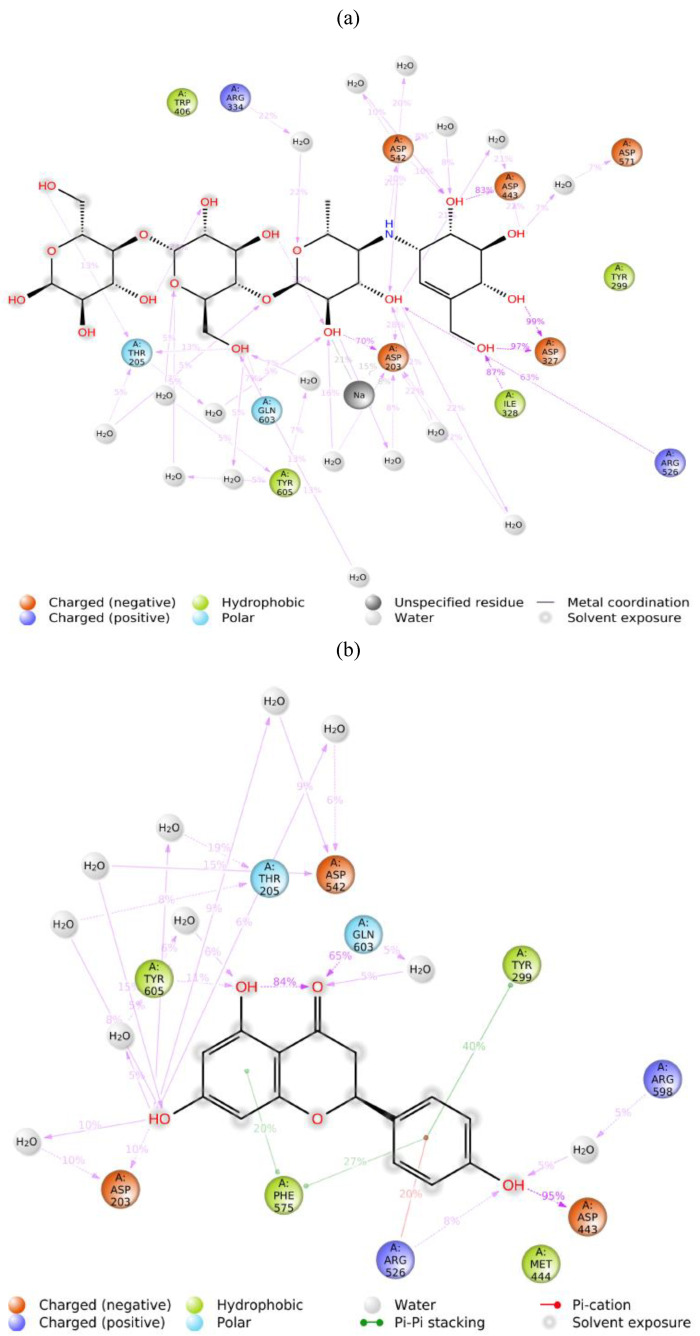
Ligand-receptor interaction percentages during 20 ns MD simulation of: (**a**) acarbose and (**b**) naringenin. Interactions that occurred for >5.0% of the simulation time are shown.

In the MD simulation, the stable complex system was analyzed for the type of protein-ligand interaction in 20 ns of simulation (Figure 5). The interaction with ASP203, ASP327, ILE328, and ASP443 was the most frequent interaction that could be maintained during the simulation. ASP203 was found to form hydrogen bonds with two hydroxyl groups of acarbose, 70% and 28%, over the 20 ns of simulation time. ASP203 and ASP327 were also found to interact by hydrogen bonding for 99% and 97% through two hydroxyl groups of acarbose. ILE328 (87%) and ASP443 (83%) were found to interact with one hydroxyl group of acarbose (Figure 6). This result suggests that those four amino acids were the critical residues that built the acarbose–α-glucosidase complex system. Unlike acarbose, naringenin participates in interactions with TYR299, ASP443, PHE575, and GLN603, through hydrogen bonding and hydrophobic interactions. The essential residues that generated the complex were ASP443, which formed hydrogen bonds with the hydroxyl group of naringenin for 95%, and GLN603 interacted by hydrogen bonding with the carboxyl groups of naringenin for 65% in the final structure after 20 ns. These could be considered key residues for the interactions. Moreover, the stability of the naringenin–α-glucosidase complex was also supported by the hydrophobic interaction of TYR299 for 40% and PHE575 for 27% and 20% by π-π stacked interactions along the benzene ring during 20 ns of simulation time.

With plant extracts being a reservoir of bioactive compounds, it has become the basic material of study for the development of new drugs. The extraction process is a crucial first step in the analysis of medicinal plants, as it is necessary to extract the desired chemical components from the plant materials for further separation and characterization. To some extent, substantial amounts of phytochemicals were extracted from the dried and ground leaf of *E. magalismontanum* by the methanol solvent. The yield of methanol fraction from crude methanol extract could be attributed to the high levels of polar phytochemicals or it being a broad-spectrum solvent capable of extracting high molecular weight compounds, such as sugars and tannins. Since plant extracts usually occur in combination with other groups of bioactive compounds or phytochemicals of varying polarities, their separation remains a considerable challenge for the process of identifying and characterizing bioactive compounds. Thus, a common practice in the isolation of these bioactive compounds is the use of different separation techniques, including solvent–solvent fractionation, column chromatography, and TLC, which were utilized to obtain the isolated compound, naringenin. The in silico assays, however, offer one of the most important and innovative approaches to target the active compound(s). This method utilizes computational techniques as a tool in predicting the interactions between a ligand with its target protein, as was the case with the isolated compound, naringenin, where its interactions with α-glucosidase enzyme were predicted and described. The traditional method for the screening of medicinal plants for the pharmacological activity of active compound(s) is an expensive process requiring energy and time. Naringenin was found to be a potent α-glucosidase inhibitor, comparable to acarbose in both in vitro and in silico assays. The results of this study showed that the ability of naringenin to inhibit the activity of these digestive enzymes is a promising source of new antidiabetic compounds.

## 3. Materials and Methods

### 3.1. Chemicals and Apparatus

Folin–Ciocalteu reagent, gallic acid, potato starch, porcine pancreatic α-amylase enzyme (type VI), Na_2_CO_3_, DPPH (2,2-diphenyl-1-picrylhydrazyl), acarbose, 3,5-dinitrosalicylic acid (DNS), sodium potassium tartrate, NaOH, rat intestinal acetone powder (a reagent for the evaluation of α-glucosidase inhibition), potassium phosphate, sucrose, TRIS [tris(hydroxymethyl)aminomethane], HCl, glucose oxidase kit (GAGO 20), ascorbic acid, Trolox, dimethyl sulphoxide (DMSO), were purchased from Sigma, Johannesburg, South Africa. Acetone, methanol, hexane, chloroform, and butanol were purchased from Merck, South Africa. The absorbance measurements were read using a Multiscan MS microtiter plate reader (Labsystems, Waltham, MA, USA).

### 3.2. Plant Materials

The leaves of *E. magalismontanum* were collected in 2018 from the Pretoria National Botanical Garden in South Africa with the GPS coordinates of −25°44′ 15.036″ S and 28°16′ 42.456″ E. The plant was authenticated by SANBI (South African National Biodiversity Institute), with voucher specimens (SMU-SM001) deposited to the Department of Biology, Sefako Makgatho Health Sciences University, Pretoria, South Africa. The leaves were dried under a shade at room temperature and milled to a fine powder.

### 3.3. Plant Extraction and Bio-Assay Guided Fractionation

**Figure 7:**
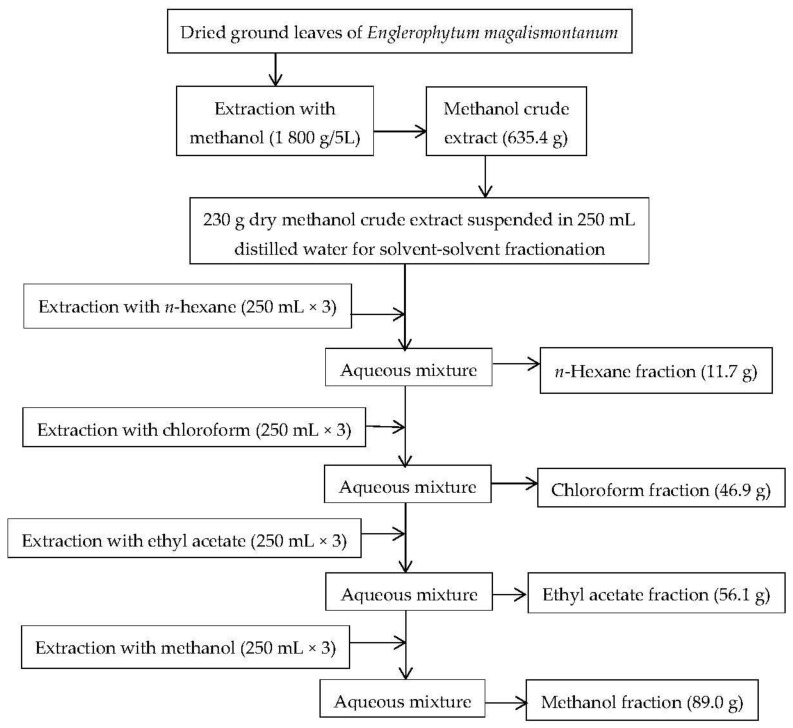
Schematic diagram for the extraction and solvent-solvent fractionation of methanol crude leaf extract.

**Figure 8:**
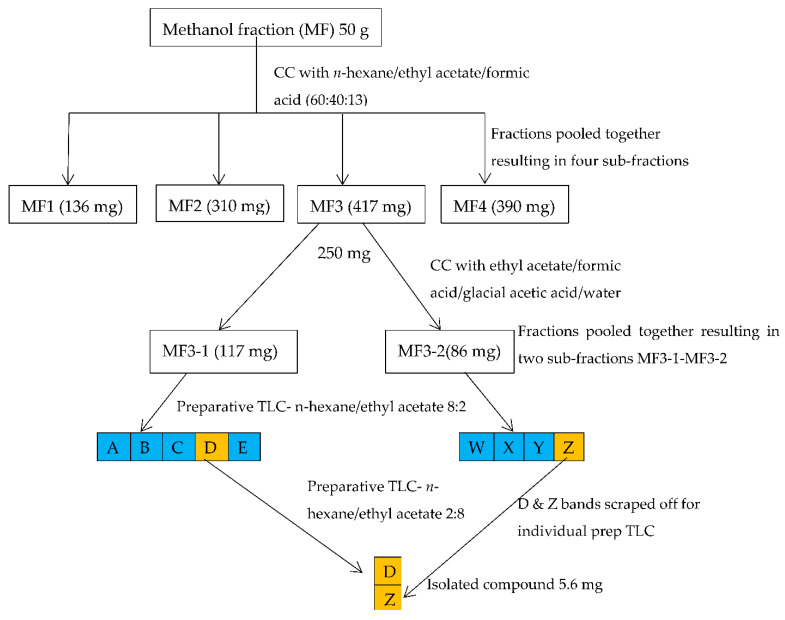
Schematic diagram of bioassay guided isolation of compound.

The pulverized *E. magalismontanum* leaf (1800 g in 5 L) was macerated with methanol for 3 days, and thereafter shaken on a Labotec shaker for 30 min. After filtration, the methanol was removed, and the residue was subjected to another extraction by soaking in methanol for 3 days. The filtrates were combined, concentrated under reduced pressure in a rotary evaporator (Rotavapor R-200 Buchi, Flawil, Switzerland), and dried under a stream of cool air to a constant weight. The resulting methanol crude extract weighed 635.4 g and was evaluated for total phenolic content (TPC), antioxidant activity, and α-amylase and α-glucosidase inhibitory activity. Part of the methanol crude extract (230 g) was subjected to solvent–solvent fractionation by suspending it in distilled water (250 mL) prior to successive partitioning by order of increasing polarity with hexane, chloroform, ethyl acetate, and methanol in the separating funnel adapted from the method of Emran et al. [43]. The fractions were concentrated by removing the solvents under pressure in a rotary evaporator to obtain a semi-dried mass which was further dried at room temperature under a stream of cool air until a fraction with constant weight was obtained (Figure 7). The resulting dried fractions, hexane, chloroform, ethyl acetate, and methanol, were evaluated for TPC, antioxidant activity, and α-amylase and α-glucosidase inhibitory activity. The bio-guided column chromatography fractionation was conducted based on the enzyme’s inhibitory activity (Figure 8). The fractionation of the most potent fraction/sub-fractions was continued until a pure compound with α-amylase and α-glucosidase inhibitory activity was obtained. The column chromatography fractionation was performed on silica gel pores with a size of 60 Å. The chromatography column was wet-packed with silica gel using methanol. Thereafter, the methanol fraction (MF) (50 g) was loaded onto the wet packed column and continuously eluted with n-hexane/ethyl acetate/formic acid (60:40:13, *v*/*v*/*v*).

Aliquots of the eluates collected were spotted onto aluminum oxide coated thin-layer chromatography (TLC) plates Merck, 60 F254 (Merck, Darmstadt, Germany), to separate into the bands that compounds present therein. The eluates displaying the same band formation on the TLC plates were pooled together, resulting in four sub-fractions MF1 (136 mg), MF2 (310 mg), MF3 (417 mg), and MF4 (390 mg). The active sub-fraction MF3 (250 mg) was subjected to silica gel column chromatographic fractionation and eluted with ethyl acetate/formic acid/glacial acetic acid/water (100:11:11:26, *v*/*v*/*v*/*v*) to yield two sub-fractions MF3-1 (117 mg) and MF3-2 (86 mg) when eluates displaying similar compound bands on the TLC plates were pooled together. These sub-fractions were each purified on a preparative TLC plate (Merck silica gel 60 × 0.5 mm thickness, Merck, Germany) and eluted with n-hexane/ethyl acetate (8:2, *v*/*v*). The bands were scraped off the plate, dissolved, and thereafter subjected to another preparative TLC and eluted with n-hexane/ethyl acetate (2:8, *v*/*v*) to yield a distinct, clear band, which was scraped and washed with ethanol to recover one pure compound. A pure compound of 3.8 mg was recovered from sub-fraction MF3-1 and the same compound of 1.8 mg from sub-fraction MF3-2. The total amount of compound obtained was 5.6 mg. The compound obtained was evaluated for α-amylase and α-glucosidase inhibitory activities while the structure was elucidated by NMR.

### 3.4. Characterization of Chemical Structure of Compound

The data of the chemical structure of the isolated compound were determined by proton and carbon (^1^H and ^13^C) distribution, ^1^H-^13^C heteronuclear single quantum correlation (HSQC) and ^1^H-^13^C heteronuclear bond multiple correlation (HMBC) [32]. The nuclear magnetic resonance (NMR) spectra were recorded on a Varian Mercury Plus 300 (Varian Inc., Palo Alto, CA, USA) spectrometer with tetramethylsilane (TSM) as an internal standard. The hydrogen (^1^H) NMR spectra were measured at 600 MHz, while the carbon (^13^C) NMR spectra were measured at 100 MHz. The NMR was performed at a constant temperature of 27 °C using the software supplied by the manufacturer. The solvent used for dissolution was deuterated chloroform (CDCl_3_). The chemical shifts (δ) were expressed in parts per million (PPM), and the coupling constant (*J*) was expressed in Hertz (Hz).

### 3.5. Total Polyphenolic Content (TPC)

The total phenolic content was determined using the Folin-–Ciocalteu method of Ragazzi and Veronese [44]. To the extract/fraction dissolved in methanol (125 µg/mL, 20 μL), 200 μL of distilled water and 40 μL of Folin–Ciocalteu reagent were added. The mixture was left to rest for 5 min at room temperature, and then 40 μL of 20% sodium carbonate was added to the mixture. After incubation at room temperature in the dark for 60 min, the absorbance was measured at 680 nm. Gallic acid was used as a standard. The TPC was expressed as the mg gallic acid equivalent (GAE) per g of dry extract.

### 3.6. DPPH (2,2-Diphenyl-1-picrylhydrazyl) Antioxidant Assay

The free radical scavenging potential of the extract was estimated using the DPPH method of Olech et al. [45]. In this assay, the relative capacity of antioxidants to scavenge the DPPH radical compared to the antioxidant activity of Trolox was measured. The crude extract or fraction was prepared in methanol to a series of concentrations (0.0625 to 1000 µg/mL), and the ascorbic acid was also prepared in a series of concentrations (0.031 to 500 µg/mL). Trolox was used as standard. In the wells of the microtitre plate in triplicate, 40 µL of methanol was added to 40 µL of extract, fraction or ascorbic acid. Thereafter, 160 µL of DPPH were added and incubated for 30 min in the dark. The absorbance was measured at 517 nm. For the blank, methanol was added instead of DPPH, and for solvent control, methanol was added instead of the extract. The percentage change in absorbance was calculated for each concentration of extract, fraction, and ascorbic acid. The antioxidant activity result was expressed as the Trolox antioxidant equivalent (TAE).

### 3.7. Enzyme Inhibitory Activity

#### 3.7.1. α-Amylase Inhibitory Assay

The α-amylase inhibition assay was conducted using the method previously discussed by Olaokun et al. [46]. In a tube, to 160 µL of distilled water, 40 µL of extract/fraction (20 mg/mL) and 200 µL of ice-cold porcine α-amylase solution (4 unit/mL) were added. After pre-incubation at 25 °C for 5 min, 400 µL of potato starch (0.5%, m/V) in 20 mmol/L phosphate buffer (pH 6.9) were added. Following incubation at 25 °C for 3 min, an aliquot of the mixture (200 µL) was removed and placed into a new tube containing 100 µL of DNS color reagent (96 mmol/L 3,5-dinitrosalicylic acid and 5.31 mol/L sodium potassium tartrate in 2 mol/L NaOH) and placed into an 85 °C-water bath. While 40 µL of the DMSO (control) replaced the plant extract for 100% enzyme activity, acarbose (1 unit/mL) was used as the positive control. After 15 min, the mixture was removed from the water bath, cooled and diluted with 900 µL of water, and then absorbance was read at 540 nm. For the blanks (negative controls), the enzyme solution was replaced with distilled water, and the same procedure was carried out as above. The α-amylase inhibitory activity was calculated from the equation; inhibition% = [(A_control_ − A_blank_) − (A_sample_ − A_blank_)/(A_control_ − A_blank_)] × 100. Where A_control_ (representing 100% enzyme activity) is the absorbance of the control well, A_blank_ is the absorbance of the blank well, and A_sample_ is the absorbance of the test extract/fraction well.

#### 3.7.2. α-Glucosidase Inhibitory Assay

The α-glucosidase inhibitory activity was determined using the method of Olaokun et al. [46]. In a test tube, sucrose (56 mmol/L, 200 µL) in 0.1 mol/L potassium phosphate buffer (pH 7) was added to 100 µL of the extracts (2.5 mg/mL) in 50% DMSO. After pre-incubation at 37 °C for 5 min, 200 µL of ice-cold freshly prepared rat intestinal α-glucosidase solution was added. DMSO (50%, 100 µL) (control) was used in place of the extract for 100% enzyme activity, whereas acarbose (0.1 mg/mL) was used as the positive control. After mixing thoroughly, the test sample, solvent, and positive control test tubes were incubated at 37 °C for 20 min, and the reaction was stopped by adding 2 mol/L of Tris–HCl buffer (pH 6.9, 750 µL). The amount of liberated glucose was determined by the glucose oxidase method using a commercial test kit (GAGO 20 Sigma, St. Louis, MO, USA) according to the instructions. The absorbance of the wells was measured at 540 nm. The α-glucosidase inhibitory activity was calculated using the equation; inhibition% = [(A_control_ − A_blank_) − (A_sample_ − A_blank_)/(A_control_ − A_blank_)] × 100. Where A_control_ (representing 100% enzyme activity) is the absorbance of the control well, A_blank_ is the absorbance of the blank well, and A_sample_ is the absorbance of the test extract/fraction well.

#### 3.7.3. Calculation of IC_50_

The concentration of the crude/fractions/compounds that inhibited 50% of the enzyme activity (IC_50_) was determined for α-amylase and α-glucosidase inhibitory activities. It was calculated from the plot of the series of concentrations versus the percentage inhibition.

### 3.8. In Silico Assay

#### 3.8.1. Ligand and Receptor Preparations

The isolated compound (naringenin) and acarbose (positive control) were used for the molecular docking simulations for α-glucosidase. The two-dimensional structure of naringenin was obtained from the PubChem database (https://pubchem.ncbi.nlm.nih.gov) (accessed on 20 June 2021) in the Molfiles Structure Data File (MOL SDF) extension. The naringenin chemical structure was built by the conversion to a three-dimensional (3D) structure and optimized by energy minimization with an MMFF94 force field using Chem 3D software, and the addition of Gasteiger charges, before being converted into a PDBQT format file with AutoDock Tools (The Scripps Research Institute, California, USA). The receptor was prepared by retrieving the 3D crystal structure of α-glucosidase from a complex with acarbose from the Protein Data Bank (https://www.rcsb.org/) (accessed on 8 June 2021) with the PDB ID: 2QMJ [47]. The receptor was optimized by removing the solvent and non-essential residues during the crystallization process using BIOVIA Discovery Studio 2020 (Dassault Systemes, Velizy-Vilacoublay, France). Acarbose, as a co-crystallized ligand, was extracted from the protein’s binding pocket to disclose the grid coordinates throughout the active site.

#### 3.8.2. Molecular Docking

The molecular docking of α-glucosidase was conducted using an AutoDock Vina (The Scripps Research Institute, San Diego, CA, USA) with a 40 × 40 × 40 Å grid box dimension and centered on −21.764 × −6.547 × −5.222 along to the binding site of *x*, *y*, *z* axes, respectively. The docking protocol was validated by the docking of the co-crystallized ligand to the binding site to evaluate the docking methodology’s accuracy. The docking protocol was valid and reliable if the docking conformation of the co-crystallized ligand was similar to that of the experimental pose with the root mean square deviation (RMSD) value < 2 Å. After validation, the naringenin was docked within the same grid box dimension on the binding sites of the receptor. The binding energy (kcal/mol) and molecular interactions were recorded and analyzed.

#### 3.8.3. Molecular Dynamics

For the molecular dynamics (MD) study, Schrödinger 2021-2 software with a Desmond module (Schrödinger, New York, NY, USA) was used. This process was commenced by generating the system of ligand-protein complexes and immersing them into SPC (simple point charge) in 10 Å water boxes. Moreover, the system was neutralized by adding counter ions that consisted of 84 molecules of Na^+^ and 62 molecules of Cl^-^. On the other hand, a salt consisting of sodium and chloride ions was added at 0.15 mol/L to make the system isotonic. This process was prepared to simulate the system in physiological conditions. The MD simulation was conducted in normal pressure–temperature (NPT) conditions with the temperature set to 300 K and the pressure at 163 kPa with optimized potentials for liquid simulations (OPLS_2005) force field for 20 nanoseconds (ns) trajectories. The recording intervals were set to 1.2 picoseconds (ps) for energy along 20 ps for trajectory.

### 3.9. Data Analysis

The experiments were performed in triplicate and repeated on three different occasions. The results were expressed as mean ± standard deviation (SD) (*n* = 9). Statistical analysis was performed where applicable by one-way analysis of variance (ANOVA) and using SPSS 20 (IBM) software (IBM, Chicago, IL, USA). Significant differences were determined by a two-tailed *t*-test, with *p* < 0.05 considered statistically significant.

## 4. Conclusions

This study investigated *E. magalismontanum* leaf extract as a potential source of phenolic antidiabetic compounds. To the best of our knowledge, the α-amylase and α-glucosidase inhibitory activity, as well as the molecular docking and molecular dynamics of naringenin, a compound isolated from *E. magalismontanum* extract, were demonstrated for the first time. Naringenin was found to be a potential α-glucosidase inhibitor in comparison to acarbose in both in silico assays. Nevertheless, further in vivo studies are required to confirm the perceived antidiabetic potential of this compound.

## Figures and Tables

**Table 1:** Antioxidant activity and total phenolic content of crude extract and fractions.

Extract/Fractions	DPPH Scavenging Expressed as TAE (Trolox Antioxidant Equivalent) µg/mL	Total Phenolic Content (mg GAE/g Dry Extract)
Crude methanol	1.66 ± 0.63 ^a^	49.78 ± 0.40 ^a^
Hexane (HF)	3.06 ± 0.77 ^a^	42.12 ± 1.53 ^b^
Chloroform (CF)	2.99 ± 2.78 ^b^	53.08 ± 1.98 ^b^
Ethyl acetate (EF)	2.24 ± 1.69 ^b^	54.41 ± 1.22 ^b^
Methanol (MF)	1.51 ± 0.66 ^a^	56.53 ± 1.94 ^b^
Ascorbic acid	0.56 ± 0.88	ND

ND—not determined, Values are means (*n* = 9) ± SD; ^a,b^ No significant difference between extracts with same value, but significant difference *p* < 0.05 between different values.

**Table 2:** α-amylase and α-glucosidase inhibition activity of crude extract and fractions.

Extract	50% Inhibitory Concentration (IC_50_) (µg/mL)	
	α-Amylase Inhibition	α-Glucosidase Inhibition
Crude methanol	16.16 ± 2.23 ^a,c^	12.25 ± 1.03 ^a,b^
Fraction		
Hexane	50.93 ± 0.25 ^b^	39.28 ± 1.02 ^a^
Chloroform	60.85 ± 0.72 ^b^	52.24 ± 1.02 ^a^
Ethyl acetate	29.18 ± 1.14 ^a^	25.20 ± 0.76 ^a^
Methanol	10.76 ± 1.33 ^a,c^	12.27 ± 1.55 ^a,b^
MF sub-fraction		
MF1	26.84 ± 1.67 ^c^	58.75 ± 1.48 ^b^
MF2	36.93 ± 1.01 ^c^	28.63 ± 1.21 ^b^
MF3	12.49 ± 0.96 ^c,d^	10.19 ± 1.04 ^b^
MF4	45.11 ± 1.36 ^c^	27.63 ± 2.02 ^b,d^
MF3 sub-fraction		
MF3-1	8.79 ± 2.23 ^d^	6.88 ± 0.79 ^c^
MF3-2	8.71 ± 1.42 ^d^	7.84 ± 2.61 ^d^
Naringenin	5.81 ± 2.14 ^d^	4.77 ± 2.99 ^d^
Acarbose (positive control)	1.24 ± 1.64	1.92 ± 0.73

Values are means (*n* = 9) ± SD; ^a,b,c,d^ No significant difference between extracts with same value, but significant difference *p* < 0.05 between different values.

**Table 3:** NMR spectroscopy data for naringenin isolated from *E. magalismontanum*.

NMR	Spectra Signal Correlations
^1^H	δ 7.37 and 6.83 ppm, (*J* = 8.6 Hz, H-2′, 6′ and H-3′, 5); δ 5.87 and 5.89 ppm (*J* = 2.2 Hz, H-6 and H-8). 5-OH δ 5.44 (^1^H, dd, *J* = 13.0 and 3.0 Hz), δ 2.70 (^1^H, dd, *J* = 17.2 and 3.0 Hz) and δ 3.15 (^1^H, dd, *J* = 17.2 and 13.0 Hz)
^13^C	δ 98.20 (C-6), δ 97.50 (C-8), δ 115.22 (C-3′ and C-5′) and δ 128.10 (C-2′ and C-6′)
HMBC	^2^J_C-H_ and ^3^J_C-H_, δ 196.28 (C-4), δ 160.10 (C-4′), δ 166.59 (C-7), δ 164.11 (C-5), δ 163.50 (C-8a), δ 129.49 (C-1′) and δ 102.19 (C-4a).

Chemical shifts and coupling constants (*J*, Hz, in parentheses) (see Supplementary Materials).

**Table 4:** Binding energy of acarbose and naringenin with α-glucosidase receptor.

Compound	Binding Energy (kcal/mol)
Acarbose	−7.9
Naringenin	−7.0

## Data Availability

The data in this study are available in the article.

## Supplementary Materials

The following supporting information can be downloaded at: https://www.mdpi.com/article/10.3390/molecules27103175/s1. Figure S1: 1H (600 MHz, CDCl_3_) NMR spectrum of naringenin; Figure S2: ^13^C (100 MHz, CDCl_3_) NMR spectrum of naringenin; Figure S3: 1H- ^13^C HMBC (600 MHz: 1H and 100 MHz: ^13^C; CDCl_3_) NMR spectrum of naringenin; Figure S4: 1H- ^13^C HSQC (600 MHz: 1H and 100 MHz: ^13^C; CDCl_3_) NMR spectrum of naringenin.

## Sample Availability

Samples of the compound were used completely for all the assays.

## Abbreviations

MMFF94	molecular modeling force field developed in 1994
PDB ID	protein data bank identifier
NPT	isobaric-isothermal ensemble
PDBQT	protein data bank, partial charge (Q) and atom type (T) format
OPLS	optimized potentials for liquid simulations
