# Supplementary material for: Molecular Docking and Molecular Dynamics Studies of Antidiabetic Phenolic Compound Isolated from Leaf Extract of Englerophytum magalismontanum (Sond.) T.D.Penn
Source: Molecules. 2022 May 16;27(10):3175. doi: 10.3390/molecules27103175 (PMC9145638; doi:10.3390/molecules27103175)
Supplement: Supplementary file 1 [file molecules-27-03175-s001.zip › molecules-1709213-supplementary.pdf]

SUPPLEMENTARY MATERIAL

NMR Spectra of naringenin isolated from *E. magalismontanum* leaf and recorded on a Varian mercury Plus 300 spectrometer with tetramethylsilane (TSM) as internal standard

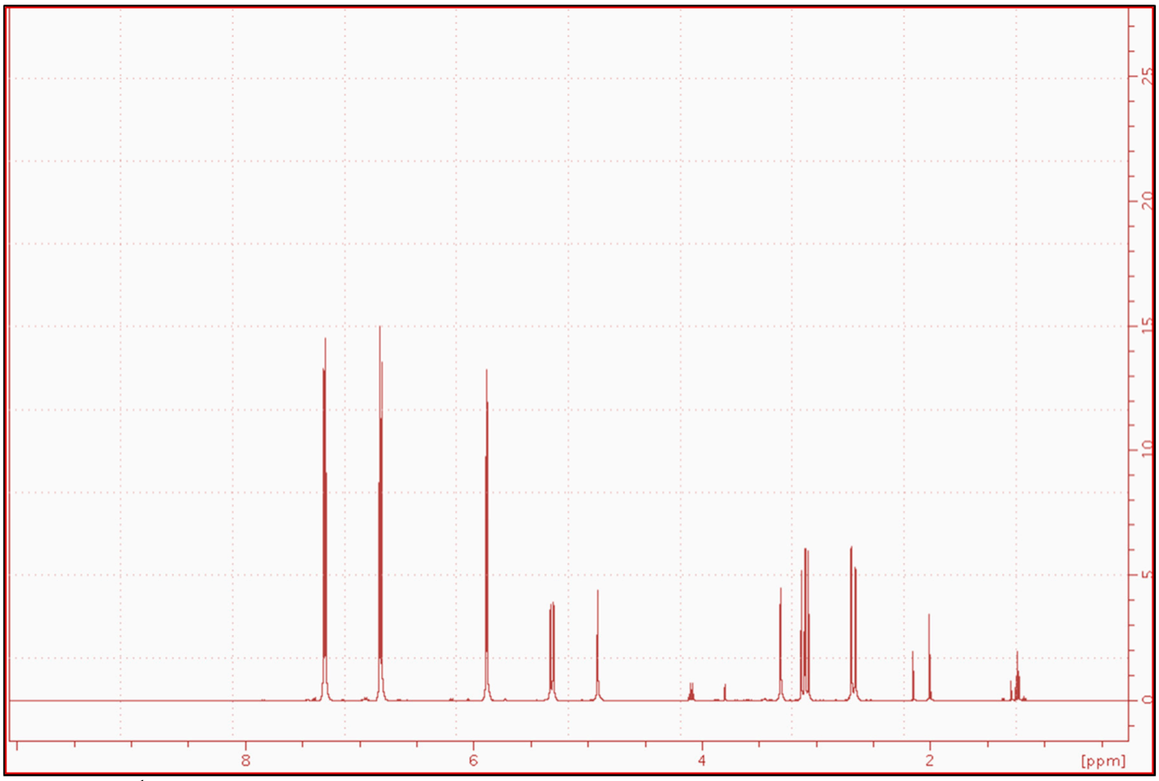

Figure S1. ^{1}H (600 MHz, $CDCl_3$) NMR spectrum of naringenin.

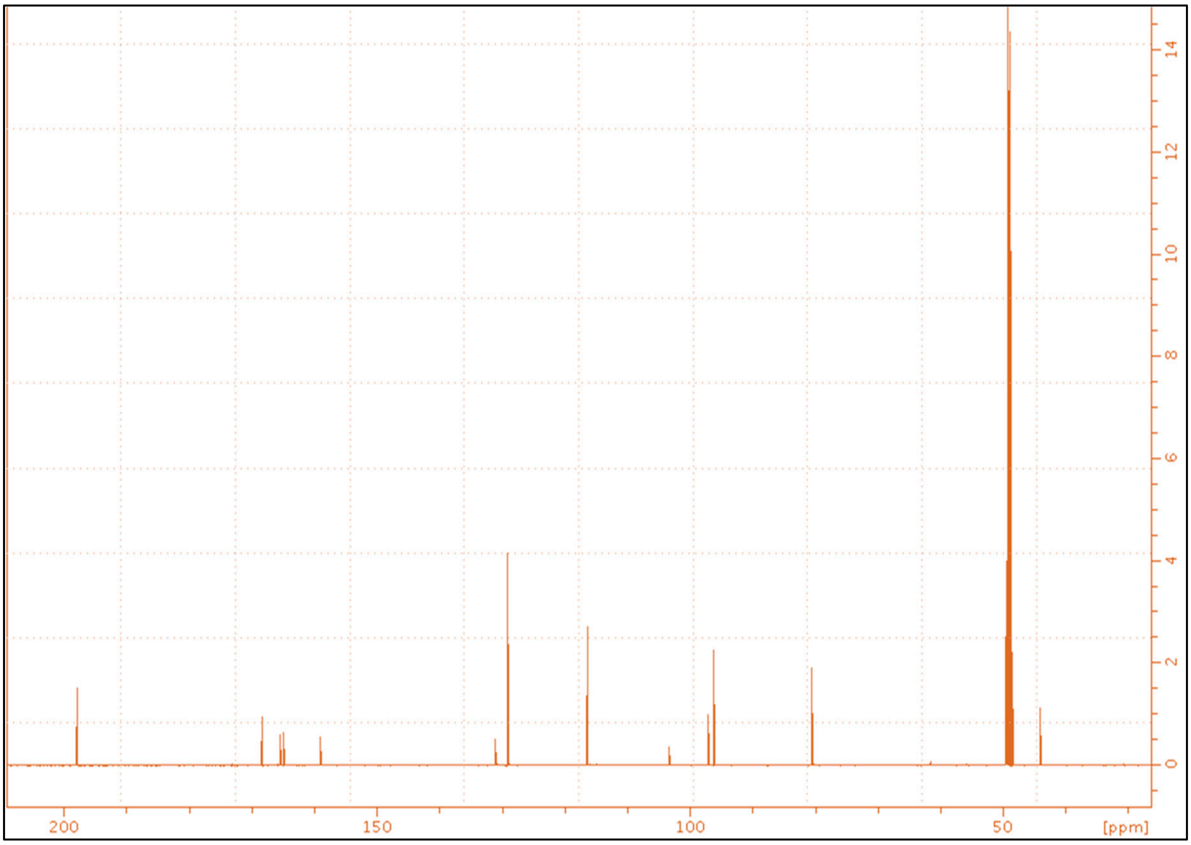

Figure S2. ^{13}C (100 MHz, $CDCl_3$) NMR spectrum of naringenin.

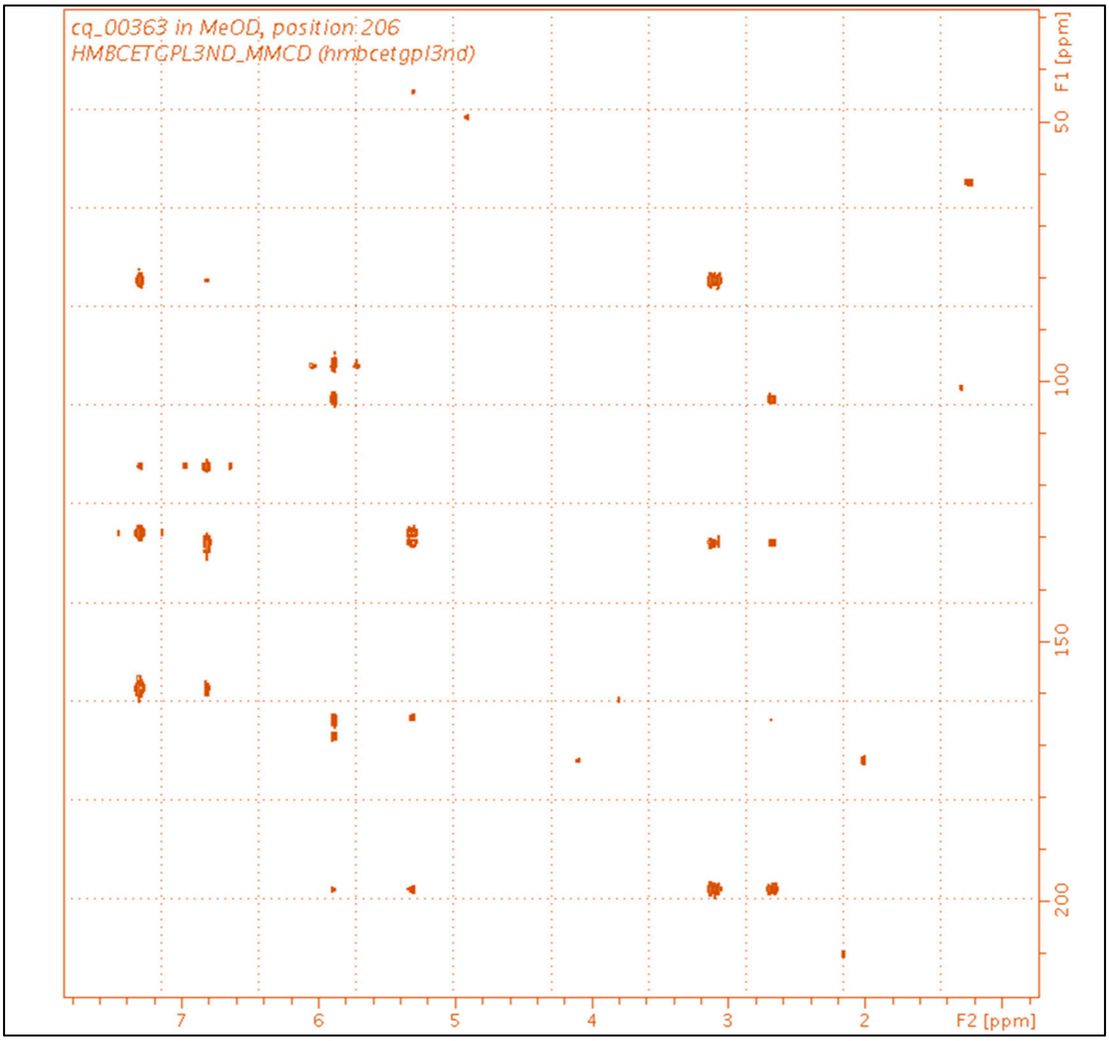

Figure S3. ^{1}H- ^{13}C HMBC (600 MHz: ^{1}H and 100 MHz: ^{13}C; $CDCl_3$) NMR spectrum of naringenin.

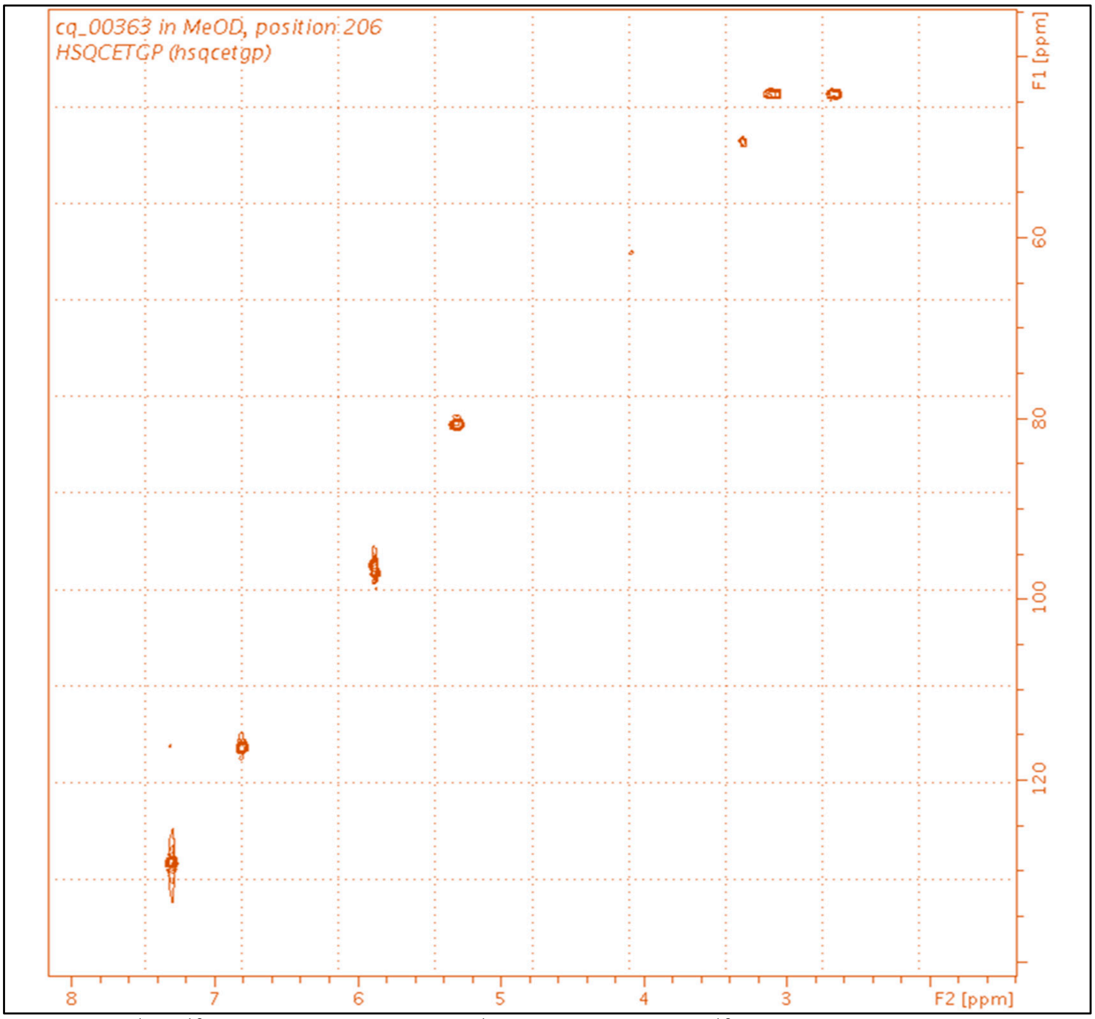

Figure S4. ^{1}H- ^{13}C HSQC (600 MHz: ^{1}H and 100 MHz: ^{13}C; $CDCl_3$) NMR spectrum of naringenin.
